# Individual Differences in Attributes of Trust in Automation: Measurement and Application to System Design

**DOI:** 10.3389/fpsyg.2019.01117

**Published:** 2019-05-21

**Authors:** Thomas B. Sheridan

**Affiliations:** Department of Mechanical Engineering, Aeronautics, and Astronautics, Massachusetts Institute of Technology, Cambridge, MA, United States

**Keywords:** trust, automation, individual differences, human-system interaction, system design

## Abstract

Computer-based automation of sensing, analysis, memory, decision-making, and control in industrial, business, medical, scientific, and military applications is becoming increasingly sophisticated, employing various techniques of artificial intelligence for learning, pattern recognition, and computation. Research has shown that proper use of automation is highly dependent on operator trust. As a result the topic of trust has become an active subject of research and discussion in the applied disciplines of human factors and human-systems integration. While various papers have pointed to the many factors that influence trust, there currently exists no consensual definition of trust. This paper reviews previous studies of trust in automation with emphasis on its meaning and factors determining subjective assessment of trust and automation trustworthiness (which sometimes but not always are regarded as an objectively measurable properties of the automation). The paper asserts that certain attributes normally associated with human morality can usefully be applied to computer-based automation as it becomes more intelligent and more responsive to its human user. The paper goes on to suggest that the automation, based on its own experience with the user, can develop reciprocal attributes that characterize its own trust of the user and adapt accordingly. This situation can be modeled as a formal game where each of the automation user and the automation (computer) engage one another according to a payoff matrix of utilities (benefits and costs). While this is a concept paper lacking empirical data, it offers hypotheses by which future researchers can test for individual differences in the detailed attributes of trust in automation, and determine criteria for adjusting automation design to best accommodate these user differences.

## Introduction

In recent years trust in automation has become an active field of research in human factors psychology and human-systems engineering. This is because user trust has been shown experimentally to play a major role in use, misuse, abuse, and disuse of the automation (Parasuraman and Riley, 1997).

This paper asserts that as automation becomes more “intelligent” users' trust of automation will increasingly resemble that of trusting another person. This is likely to result in increasingly greater individual differences among human trusters, as well as the differences in computer-based automation itself, the objects of the user trust. Such trust has already been shown to depend on many different objective attributes of the automation's performance such as capability and statistical reliability. In the future, with increasing computer “intelligence,” sociological considerations of culture and morality will also become significant factors of trust in automation. The paper details how these attributes apply.

Thus, future automation may itself develop a reciprocal capability of modeling trust in its user and modification of its behavior toward the human user as a function of that trust. This reciprocity can be thought of as a cooperative/competitive game between human and computer agents.

The term *automation* in this paper refers to the hardware and software systems that enable any or all of sensing, analysis, memory, decision for action, and implementation of that action in order to better achieve a given desired result. The term *intelligent* when applied to automation refers to incorporating the increasingly sophisticated heurists and algorithms of artificial intelligence (e.g., neural net “deep learning,” pattern recognition, etc.) beyond the feedback control techniques of traditional automation.

*Trust* in some particular automation is a human's propensity to submit to vulnerability and unpredictability, and nevertheless to use that automation, as measured by intention expressed in speech or writing, or by measurable bodily actions to actually use the automation. Reciprocal trust in a particular human user by advanced automation will probably take the form of computer database regarding past interactions with that user and/or computer-based decision/prediction rules.

## Past Research on Trust in Automation With Focus on the Meaning of “Trust”

Much of the literature on trust in automation has focused on issues such as trust calibration: whether the truster is overtrusting (is too complacent) or undertrusting (compared to statistical reliability justification) especially with respect to choice between automatic or manual control (see Muir and Moray, 1996; Parasuraman and Manzey, 2010). Experiments on trust tend to be conducted in specific settings and concerns, such as fidelity of alarms and advisory displays in aircraft, hospitals, nuclear plants, robots, etc. Miller (2004) emphasizes that trust is especially important for adaptive automation.

Lee and See (2004) provide an extensive review and a qualitative model or framework of trust in automation. There are other efforts to model trust and achieve some level of quantification. Gao and Lee (2006) utilize decision field theory to model sequential decisions in a dynamic supervisory control context. Chiou and Lee (2016) use a joint task mircro world to study the cooperative behavior of agents and resource sharing. Hoffman et al. (2009) also model trust in a dynamic human-computer situation. Such models tend to start with data on whether the truster makes trust/distrust binary decisions or specifies a degree of trust on a subjective scale, and then the models perform quantification based on those human actions. Sheridan (2019) shows how existing models for signal detection, calibration of judgments of statistical parameters, or internal model-based techniques such as presently used on control engineering, can be applied to modeling trust.

Regarding factors that define trust, Hancock et al. (2011) devised an extensive scale of trust-related factors and antecedents, and obtained correlations of judgments on how these relate to differing classes of robots. Hancock et al. (2011) also provides a meta-analysis of trust related factors within a robot operating context. Lyons et al. (2011) conclude from a factor analysis experiment that trust and distrust might be orthogonal properties and are independent from judged validity of trust in automation, what they call “IT suspicion.” Hoff and Bashir (2015) review 101 papers that include 127 studies on trust in automation with the aim of sorting out factors that they then categorize with respect to the truster's disposition, the situation and the aspect of learning. They provide a useful taxonomy of design recommendations based on various authors' findings that include the following design features: appearance, ease-of-use, communication, transparency, and level of control. Mayer et al. (1995) define trust in a commonsense way as a “willingness to be vulnerable to the actions of a machine based on expectation that the machine will perform a desired action.”

There is a large related literature on computer etiquette: display and control options that are pleasing to the user to interact with and are affectively desirable. Parasuraman and Miller (2004) provide reasons why etiquette is important to enhance trust. Dorneich et al. (2012) also emphasize the particular importance of etiquette for adaptive automation. Nass and Moon (2000) offer appropriate rules of etiquette and trust to enable automatic systems to be considered teammates.

In some papers trust has been defined as a purely subjective property of the human user of automation, with trustworthiness being an objective function of the automation itself. Other papers regard trustworthiness simply as a subjective judgment of trust.

Trust has been defined in many different ways in the literature, and this paper will try to explicate these ways further, both with regard to the trust vs. trustworthiness aspect and especially with regard to the meaning of trust as computers become more “intelligent,” as defined above.

## Objective Trust/Trustworthiness Attributes

In one of the earliest papers to address the trust in automation issue Sheridan (1988) discusses the nature and importance of trust in military command and control systems, proposing a set of seven key properties.

Noting the overlap between trust attributes suggested by various authors I will here make use of the Sheridan (1988) set which has a more detailed breakdown. I assert that these may be called *objective* attributes, since they are conceivably measurable by objective means, and can be distinguished from subjective (affective) attributes to be proposed in the next section:

1. ***Statistical reliability*** (lack of automation error)
2. ***Usefulness*** (ability of the automation to do what is most important, for example in trading benefits and costs)
3. ***Robustness*** (ability and flexibility of the automation to perform variations of the task)
4. ***Understandability*** (transparency of the automation in revealing how and why it is doing what it is doing)
5. ***Explication of intent*** (automation communicating to the user what it will do next)
6. ***Familiarity*** (of the automation to the user based on past user experience)
7. ***Dependence*** (upon the automation by the user as compared to other ways of doing the given task).

The first five objective attributes are trustworthiness properties of the automation, while the last two are trust attributes of the user. It is proposed that all are applicable to automation in general.

Muir and Moray (1996) posed six related attributes: (1) reliability; (2) dependability; (3) competence; (4) predictability; (5) faith; and (6) responsibility. It can be argued that these terms mostly agree with those of Sheridan (1988): (1) Both include predictability. (2) Dependability is in many ways another way to characterize usefulness. (3) Competence is almost a synonym for robustness. (4) Predictability is akin to understandability, since users can predict future automation actions if they can understand how it works. Faith is abetted by both familiarity and by knowing the automation's intent. A perception of the automation's responsibility is engendered by both the user's familiarity with it and dependence upon it. (One can always argue that the Muir and Moray terms have different meanings that do not quite correspond to those of Sheridan).

Other authors have discussed attributes of automation that relate to the above taxonomy. In particular Christofferson and Woods (2002) discuss *observability* (shared representation of problem state and the current and planned actions of the automation, which bears on both (4) *understandability* and (5) *explication of intent*. They also discuss *directablity* (ability of the human supervisor to exercise control over the automation, which bears on (2) *usefulness*.

The seven attributes of trust detailed above can be said to be objective in the sense that operational measures can be derived to characterize the level of trustworthiness or trust. It is important to distinguish objectively measurable trustworthiness of the automation from trust by the individual human user, different perspectives that are often confused. Sheridan (2019) discusses this further and provides a graphical distinction between the two.

## Subjective (Affective) Trust/Trustworthiness Attributes of Advanced Automation Based on “Intelligent Automation,” Analogous to Properties of Human Morality

Haidt (2012), who calls himself a morality psychologist, has proposed six attributes (he calls them “foundations”) of human moral behavior:

1. ***Care/harm*** (operating out of kindness and concern, never harming others)
2. ***Liberty/oppression*** (enhancing opportunities for others, not constraining them)
3. ***Fairness/cheating*** (acting in a way that the community considers to be impartial and honest, not taking advantage of others)
4. ***Loyalty/betrayal*** (being faithful to commitments and obligations)
5. ***Authority/subversion*** (properly exercising power and control given by others, not subverting same)
6. ***Sanctity/degradation*** (upholding sacredness of the dignity and rights of others as human beings, not deprecating them).

These can be thought of as continuous scales, with the first term for each pair being a generally desirable property of human behavior and the second term being an undesirable property. This is the stuff of moral psychology and sociology. One cannot assert that these are fully independent of one another (orthogonal), but they approach what can be managed within the complexities and the linguistic and measurement constraints of those fields.

Haidt discusses in detail how individual differences with respect to these attributes play out in human interactions of all kinds. Human intuitive response is generally acknowledged to be immediate as contrasted to much slower judgments based on deliberative consideration (Kahneman, 2011). Thus, intuition is typically at a very different point in the six-dimensional space of these attributes, and plays a key role in the confirmation bias so well-established in human decision-making (Nickerson, 1998). Apart from Kahneman's “thinking fast vs. thinking slow” difference there is the question of rationality vs. rationalization. (Haidt discusses how Plato's brother Glaucon argued with Socrates that people adopt characteristics based on fear of getting caught and/or building their reputations rather than true altruism). Haidt shows how the mentality of what he calls a WEIRD demographic (western, educated, industrialized, rich, and democratic) operates with a very different weighting of these attributes compared to people in non-western societies where family and tradition are the foundation of values. Haidt also shows how the attribute of care/harm is most sacred to political liberals, while that of liberty/oppression is dominant for political libertarians, and for social conservatives all attributes are more or less evenly weighted. These factors correlate with large individual differences in what people regard as the bases of morality and hence how they behave with respect to one another.

So what do these individual differences in regard to how people judge one another have to do with trust in automation? It is clear that automation is rapidly becoming more complex, more “intelligent,” more robust in terms of what we are asking it to do for us, and hence more variable and less predictable in many ways, due largely to the limits of users' understanding of what makes it tick. For any particular type of automation and/or software only a narrow subset of users will actually be competent to understand. (In the case of the huge neural nets that are the basis of “deep learning” in artificial intelligence, understanding how the automation arrived at its decisions and actions is essentially not even possible!). Therefore, my premise here is that as automation becomes more human-like, even “multi-cultural,” large individual differences in attributes normally associated with human affect and morality will occur, and knowledge of these can be applied usefully to automation. This does not mean that the more objective attributes listed under Past Research no longer apply. It is rather that we have a fuller set of considerations by which to evaluate trust in and trustworthiness of automation. Fulfillment of the “automation morality” objectives is clearly becoming more relevant as automation becomes more sophisticated in sensing, memory and decision capability. Individual differences in trust as measured and modeled by increasingly intelligent automation can serve as triggers for adaptive change in the human-automation interaction (Feigh et al., 2012).

## Applying the Haidt Attributes to Intelligent Automation

Consider now how Haidt's subjective (affective) attributes apply to trust in automation. These can be regarded as continuous scales:

1. ***Care/harm***. Degree to which the automation cares about its user based on its understanding of the user's desired task objectives and the abilities or constraints of the user in supervising the automation. Insofar as possible it will take account of the user's speed of response, user preferences and programming errors, etc. It will cause no harm to him or her.
2. ***Liberty/oppression***. Flexibility of the automation in allowing the user to program in various ways, provide different displays and interaction/control options to suit the user preference, and insofar as possible be resilient when the user makes procedural errors, giving advice on how to correct errors and simplify the interaction. This might be a subjective user reaction to lack of the *directabilty* property as defined by Christofferson and Woods (2002).
3. ***Fairness/cheating***. The degree to which the automation is consistent and will not demand more speed, knowledge, or programming skill than can be expected from the user. It will provide feedback to the user when some instruction or request is beyond its (the automation's) understanding or capability. It will not take actions that are in conflict with the user's apparent intentions unless user safety is at significant risk. In the latter case it will explain why it deviated.
4. ***Loyalty/betrayal***. The degree to which the automation will record its interaction with each user so as to remember, anticipate and conform to the user's style of supervision. Insofar as possible it will anticipate user demands and be ready to perform for the user when called upon.
5. ***Authority/subversion***. The extent to which the automation will perform as requested, taking decisions, and actions based on knowledge sufficient for the assigned task. It will optimize with respect to speed, accuracy and resource use in consideration of the user's objectives, or otherwise operate on the basis of transparent default objectives.
6. ***Sanctity/degradation***. How much the automation exhibits politeness in visual display and speech communication with the user. Feedback will be provided at a level requested by the user or implied by user language and control style. Such communication will be clean, orderly and as straightforward as possible. It is noted that politeness in human-computer exchanges is already becoming a topic of active research (Meyer et al., 2016).

The user's subjective judgment with respect to fulfillment of these criteria would constitute the affective component of the user's trust.

### Which Attributes Are Most Important?

One might wonder which of the above attributes are more important, and which are less important, considering both the seven objective ones and the six subjective ones. The answer is that it totally depends on context. For example, in a nuclear power plant the automatic shutdown mechanism that drops control rods into the reactor to stop the fissile reaction when certain programmed conditions are met is a huge commitment in terms of both safety and economics, and must be done instantly and without human operator intervention. So *statistical reliability* for this rarely used operation is critical, and essentially all the other trust attributes that make for better objective or subjective human interaction are essentially irrelevant. At another extreme consider an automatic kitchen gadget such as an expresso coffee maker. If it works fine but in terms of color and gaudy design the subjective attribute of *sanctity/degradation* (aesthetics) may become the critical reason it is rejected and returned to the store. Or if, after considerable effort in coping with poorly written instructions or labeling, the user cannot figure out how to make it work and returns it in disgust—that is a lack of the objective attribute *understandability*. Or, a neophyte user may upon first use scald her fingers, and reject the coffee maker because of subjective *care/harm*. Any one or few trust attributes can become most important, whatever the complexity or level of sophistication of the automation.

## Reciprocity: Modeling of Trust of the Human User by the Computer, and its Applications

The descriptions of the automation trustworthiness attributes described above imply that the automation can record its interaction with and model its own trust in the human user. Exactly how the trust database for each individual user is constructed is itself a major research need. The automation trustworthiness attributes listed above could surely provide a starting point.

Thus, after sufficient interaction with the user, the automation can build up what has come to be called an *internal model* of the user's trustworthiness. This might take the form of a Bayesian IF-THEN contingent probability estimation of what the user is likely to do given the current circumstances. This would necessarily constitute a large computer-based state-space representing combinations of machine sensory and action data. Modern-day computer memory is easily sufficient for such a task at relatively low cost. The research challenge is what to record, what to decide about human user trustworthiness, and how the automation should modify its own behavior in response, in order to become a good team member. The Kalman (1960) estimator internal model already so common in continuous dynamic control systems provides a hint (see qualitative explanation in Sheridan, 2017).

What actions might the computer take on the basis of such a trust model of the user? Surely some actions may be taken to benefit the user, such as offering suggestions or encouragement with regard to which of several display or control modes might be easier or quicker. There may be need for some actions to be coercive—just rendering some display or control options inoperable, either to simplify things for the given user or to prevent damage to the automation or wasted time/energy. These actions may be based on long term statistical evidence of the given user's behavior, or on a short-term prediction that the particular user may be headed for trouble. It is an open question for research as to whether the computer should convey back to the user the reasons for such actions. Etiquette research suggests that users prefer to understand not only what the computer is “thinking” but also why.

**Figure 1:**
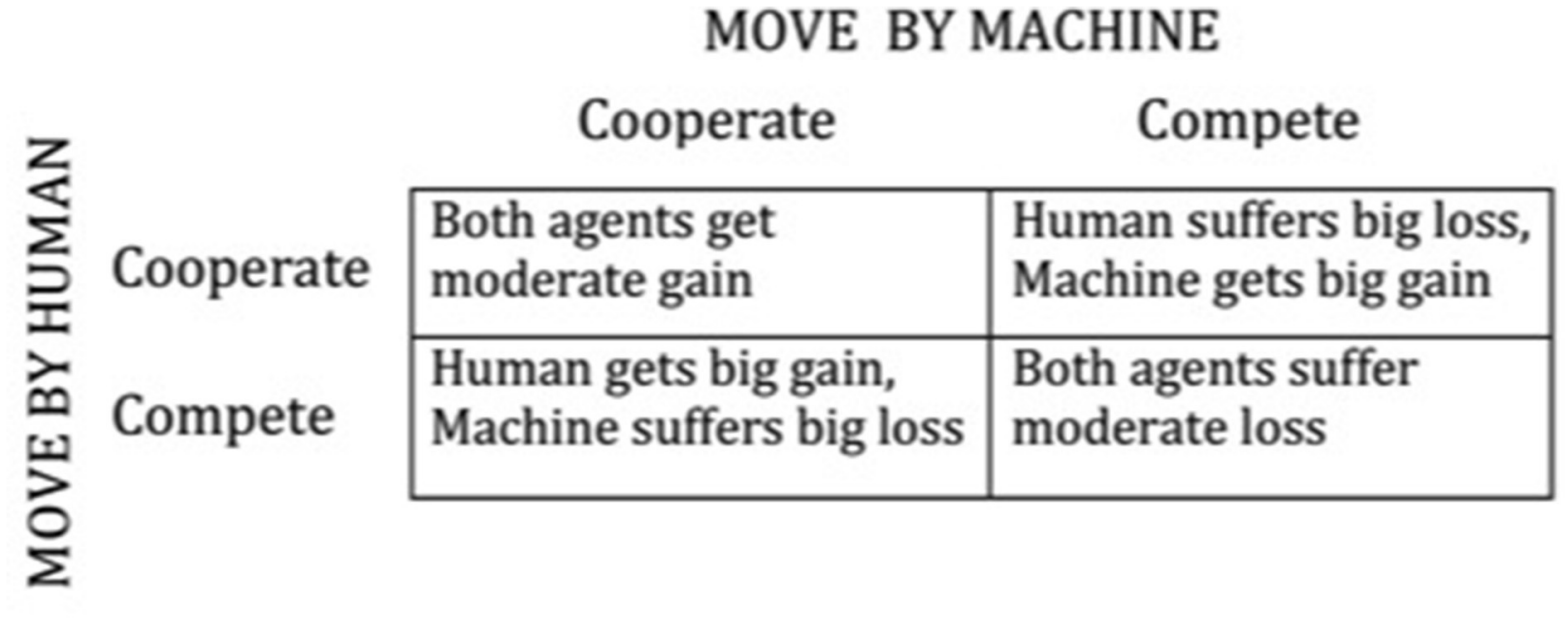
Simple 2 × 2 matrix of payoffs for prisoner's dilemma game.

Ultimately the nature of trust reciprocity might evolve into a situation representable by a formal game, where each of two agents chooses among two or multiple alternatives and the resulting payoff to each party is either a joint function of continuous response of human and machine, or a discrete payoff matrix. Most of game theory (for example in modeling business interactions, or warfare) is competitive, each agent endeavoring to maximize its own gain, often at the expense of the other agent. Sometimes the payoff matrix allows for cooperation between agents, where each agent trusts the other agent and both choose among alternatives so as to maximize a total gain (or minimum loss), which they can agree to share. However, the payoff matrix can allow for what is commonly called a “prisoners' dilemma” (named after a situation where one party trusts that the other will agree on a joint excuse that will minimize their joint penalty, but the other cheats, thus resulting in a best outcome for himself but incurring significant harm to the other. Mutual trust is mutually beneficial so long as one agent does not take the other for a sucker. The payoffs described in words in Figure 1 characterize the latter relationship. One would hope that future smart computers can be designed to avoid such a situation.

### Examples

Two examples might be helpful to understand the possibilities for the mutual trust/distrust interaction between user and advanced automation when the automation embodies a database/model of previous user trustworthiness. Consider a warning light that signals an almost empty automobile gas tank. If the driver repeatedly keeps driving so as to come perilously closer to empty, the set point for the light to come on could be adjusted to leave a bit more gas in the tank. Similarly, if the driver is recorded to have repeatedly forced the car's automated cruise control to activate the brakes and override the accelerator pedal, the speed set point can discipline the driver by imposing an even slower braking activation and/or can give an audible warning.

At a more sophisticated level, consider an aircraft flight management system, the advanced autopilot system that is in most commercial aircraft. The pilot programs navigation waypoints using letter codes and issues other instructions using a keyboard. If the computer finds the commands are un-interpretable, the trustworthiness database/model can elicit “Do you mean waypoint X?” based on its knowledge of likely pilot intentions. Other pilot commands may have interpretable meaning, but only make sense in operational contexts different from those for the current phase of flight. A computer database of pilot actions combined with data on current flight phase and normal expectations could provide corrective suggestions and advice, as well as make recordings useful for post-flight analysis.

Analysis of such a human-automation interaction might take the form of recording iterative and reciprocal moves by human and automation, with a judgment of costs and benefits of each successive action and how they interplay (a running “game matrix” as mentioned above).

## Increasing Diversity in both Technologies and People's Reaction

With time, many forms of advanced automation will emerge, sharing domestic and international markets, and sharing common physical spaces such as homes and roadways with older and simpler varieties of automation. This is already true with home appliances, highway vehicles, aircraft, medical instruments, and communication devices. Education, socioeconomic status, age and other demographic factors of human users will obviously influence human understanding, acceptance and use of new technology. The two sets of trust attributes proposed above will help discriminate individual differences between automation users in different application settings. Such individual differences will pose a serious challenge to capitalizing on the full potential of advanced automation and even create safety and fairness issues in mixed-use contexts (e.g., mixes of conventional and self-driven vehicles on the highway). The objective and subjective attributes described above will figure in the arguments of both proponents and opponents of introducing the new technologies. Trust will help determine what gets built, used, or replaced.

## Implications for Research and Design for Adjusting to Individual Differences

Proposed above are two sets of attributes applicable to trust in automation, some of which are measurable by objective means and some of which would require subjective scaling. Several types of follow-on research are proposed relating to individual differences in trust.

The attributes are proposed here as intuitively independent and comprehensive sets, but surely there is some correlation between the meanings of the terms for different subjects and different application contexts. In any case, by parsing the meanings of “trust” for different automation users there is opportunity to adjust automation to comply with individual differences between users. Below are suggested measures and design applications to that end.

### Proposed Follow-on Empirical Measures

1. Within subjects determine rank ordering and/or cardinal weighting of the attributes with respect to general importance and/or with respect to more specific criteria, such as those most frequently occurring in experience with particular automation or those most related to safety. This should be done separately for the objective attributes and the subjective attributes.
2. Across subjects calculate averages and variability of the numbers resulting from (1) for each attribute separately within the proposed objective and subjective groupings.
3. Within subjects determine ratings of the degree of meaning similarity between attributes, using a matrix including both objective and subjective attributes on each axis (with identity cells deleted).
4. Across subjects calculate averages and variability of the numbers resulting from (3).

It is likely that if the above measurements were made with a broad representation of subjects (with respect to age, gender, socioeconomic status, education, etc.) and consideration of “automation in general” there would be great variability between subjects as to the importance of the different attributes of trust. Therefore, results might be meaningless with regard to application to any particular type of automation. For that reason it would seem to make more sense that such measures be made for subject populations that are expected users of particular types of automation. Examples might be airline pilots, anesthesiologists, construction workers, users of home computers, users of washing machines, or prospective purchasers of self-driving cars. For each subpopulation there will still be individual differences attributes in the weighting of trust attributes, based on experience, education, etc. but these differences will have meaning with respect to the particular type of automation.

### Proposed Application of the Above Measures for System Design

1. What expected users indicate as the most important attributes of trust for particular types of automation will suggest design criteria for modifications or for original design of new systems. For example, with the objective attributes, heavy weighting on reliability suggests that designs should ensure reliability even at the cost of other factors. Weighting on usefulness and/or robustness suggests concern that automation become too special purpose, not designed for a broad enough scope of tasks. Weighting on understandability and/or explication of intent suggests that users may have had difficulty understanding what the automation is doing, why it is doing that, and what it is about to do. The latter problem was deemed especially important to airline pilots when they had to transition to new highly automated flight management computers (autopilots). Weighting on familiarity suggests that users take time to read the instructions, or give the new car or other system a good tryout before purchase, or make sure the users ask all their questions or know how or where to get them answered.
  For the subjective attributes, heavy weighting on care/harm suggests emphasis on design for safety. Weighting on loyalty/betrayal or authority/subversion suggests a need to ensure that what the user intended to program into the automation is what the automation understands that it is expected to do, and that it will provide feedback to put the user at ease. Weighing on sanctity/degradation suggests that the user values politeness and simplicity on the automation's part in the interaction with the user.
2. As suggested in the preceding section, future “intelligent” computers will have the capability to record the interactions between particular users over time, and make adjustments in the operation to accommodate those individual differences. For example, there already are built in adjustments that users can make in computer-based displays, means to code or key-in commands. Users may have their favorites. So, after recognizing particular users and their preferences, the automation can suggest which display or control modes are quicker (e.g., special key combinations) or which are easier to understand (e.g., displays with less abstraction and more pictorial representation, commands requiring typing, or speaking full words). It is also possible that the intelligent automation will be able to constrain the user in some way to prevent misuse or abuse of the automation. For example, some aircraft already prevent the pilot from making certain maneuvers that are likely to stall the aircraft (prevent excessive pitch up or low speeds in the thin air at high altitudes). Other constraints could be added based on computer-observed behavior (e.g., to prevent sudden pitch up on takeoff that might cause the tail to drag on the runway).

## Conclusions

1. Objectively measurable attributes of automation trustworthiness and human trust in automation are proposed.
2. Attributes of human morality proposed by Haidt (2012) are applicable as subjective (affective) trust criteria of advanced (“intelligent”) automation.
3. The increasing diversity of advanced automation, and the variety of human reactions as measured by the proposed objective and subjective trust metrics, will correlate with large individual differences in human acceptance and user capability with advanced automation.
4. Specific research to identify weightings and meaning similarities between trust attributes is suggested, with sampling for different automation contexts and classes of users.
5. Insofar as advanced automation has the capability to record detailed interactions with different users, it can build its own internal models of trust in given users.
6. These models can be used to make the automation adapt to different users: to either assist the user or to prevent resource waste or damage to the automation. Such computer-based internal models of external physical systems have precedent in dynamic control engineering practice.
7. Interaction between a human and a machine, each having an internal trust model, can be represented as a formal game between agents, with outcomes based on a payoff matrix (or other form of objective function).

## Author Contributions

The author confirms being the sole contributor of this work and has approved it for publication.

### Conflict of Interest Statement

The author declares that the research was conducted in the absence of any commercial or financial relationships that could be construed as a potential conflict of interest.
